# Medical costs and hospital utilization for hemophilia A and B urban inpatients in China: a national cross-sectional study

**DOI:** 10.1186/s12913-022-07626-x

**Published:** 2022-02-19

**Authors:** Zhengwei Huang, Stephen Nicholas, Yong Yang, Xiaoping Chen, Elizabeth Maitland, Yong Ma, Xuefeng Shi

**Affiliations:** 1 School of Management, Beijing University of Chinese Medicine, Beijing, China; 2 Australian National Institute of Management and Commerce, Eveleigh Sydney, NSW Australia; 3 School of Economics and School of Management, Tianjin Normal University, Tianjin, China; 4 Guangdong Institute for International Strategies, Guangdong University of Foreign Studies, Guangzhou, China; 5 Newcastle Business School, University of Newcastle, Newcastle, Callaghan Australia; 6 Medical Device Regulatory Research and Evaluation Centre, West China Hospital, Sichuan University, Chengdu, China; 7 School of Management, University of Liverpool, Chatham Building, Chatham Street, L697ZH Liverpool, England; 8 National Institute of Healthcare Security, Capital Medical University, Beijing, China

**Keywords:** Hemophilia, Medical cost, Hospital utilization, Urban China

## Abstract

**Background:**

Hemophilia care in mainland China has been greatly improved since the establishment of the Hemophilia Treatment Center Collaborative Network of China (HTCCNC), and most of drugs for hemophilia have been covered by basic medical insurance schemes. This study assesses whether medical costs and hospital utilization disparities exist between hemophilia A and hemophilia B urban inpatients in China and, second, whether the prescription of coagulation factor concentrates for hemophilia A and hemophilia B inpatients was optimal, from the third payer perspective.

**Methods:**

We conducted a retrospective nationwide analysis based on a 5% random sample from claims data of China Urban Employees’ Basic Medical Insurance (UEBMI) and Urban Residents’ Basic Medical Insurance (URBMI) schemes from 2010 to 2016. Univariate analysis and multiple regression analysis based on a generalized linear model were conducted.

**Result:**

A total of 487 urban inpatients who had hemophilia were identified, including 407 inpatients with hemophilia A and 80 inpatients with hemophilia B. Total medical cost for hemophilia B inpatients was significantly higher than for hemophilia A inpatients (USD 2912.81 versus USD 1225.60, *P < *0.05), and hemophilia B inpatients had a significantly longer length of hospital stay than hemophilia A inpatients (9.00 versus 7.00, *P < *0.05). Total medical costs were mostly allocated to coagulation factor products (76.86-86.68%), with coagulation factor cost of hemophilia B significantly higher than hemophilia A (*P < *0.05). Both hemophilia cohorts utilized greatest amount of plasma-derived Factor VIII, followed by recombinant Factor VIII and prothrombin complex concentrates.

**Conclusions:**

Patients with hemophilia B experienced significantly higher inpatient cost, coagulation factor cost and longer length of hospital stay than patients with hemophilia A. Our findings revealed the suboptimal use of coagulation factor concentrate drugs and a higher drug cost burden incurred by hemophilia B than hemophilia A inpatients. Our results call for efforts to strengthen drug regulatory management for hemophilia and to optimize medical insurance schemes according to hemophilia types.

## Background

Hemophilia is a rare hereditary disease linked to abnormalities in the X**-**chromosome [1]. The presence of specific genetic mutations results in an inability to generate the coagulation material essential to stop bleeding, leading to disability, pain and early death [2]. Hemophilia A caused by deficiency of clotting Factor VIII and hemophilia B caused by deficiency of clotting Factor IX are the two main types of hemophilia, accounting for the great majority of the disease [1]. Globally, the incidence rate of hemophilia A among male population is 1/5000, and that of hemophilia B is 1/30,000 [2], which is independent of race and geographical location [3]. In a meta-analysis, the prevalence of hemophilia among males was estimated to be 5.5/100,000 in mainland China [4]. According to the latest information from China’s National Hemophilia Registration System, there were altogether 16,083 patients with hemophilia A and 2447 patients with hemophilia B registered in mainland China in 2019 [5].

Providing high-quality health care to hemophilia patients is an important healthcare objective in China, with the government and the healthcare system jointly providing accessible hemophilia care. Strengthening the healthcare system and enacting related hemophilia guidelines has been a significant government strategy for improving health outcome for hemophilia patients. Hemophilia care capacity has increased continuously since 2004, when the Hemophilia Treatment Center Collaborative Network of China (HTCCNC) was established. The HTCCNC, comprises 120 centers throughout the country, playing a crucial role for hemophilia care provision [5, 6]. Affordability and accessibility for hemophilia care have also been improved remarkably by the expansion of universal medical insurance coverage, which provides partial financial support for inpatient and outpatient treatment, drugs and diagnostic expenses. Covering over 95% of the urban population, the Urban Employees’ Basic Medical Insurance (UEBMI) covers urban workers, and the Urban Residents’ Basic Medical Insurance (URBMI) covers the unemployed, retired, children, elderly and students. The major as well as the most effective drug treatment products for hemophilia depends on coagulation factor concentrates. The plasma-derived factor VIII coagulation factor concentrates (pdFVIII) [7] have been covered by the UEBMI and URBMI since 2004. In 2009, the recombinant factor VIII clotting factor concentrates (rFVIII) and prothrombin complex concentrates (PCCs) were added to the lists of the two basic medical insurance schemes [8]. In 2017, drug coverage was further expanded, with recombinant factor IX clotting factor concentrates (rFIX) and recombinant activated factor VII (rFIIa) partially paid by insurance [9]. By 2017, almost all the coagulation factor concentrates products for hemophilia have been covered as co-payments between the insurance funds and patients, which means patients with hemophilia have access to hemophilia treatment through universal health insurance.

In spite of the joint government—healthcare system efforts to treat hemophilia, the hemophilia care level in China lags behind developed countries [6]. In a comparative investigation of quality of life between China and nine other countries, Sun et al. found that Chinese hemophilia patients received less prophylaxis treatment, faced greater difficulty in obtaining replacement factor products and were vulnerable to more annual bleeds [10]. Previous research also reveals that patients with hemophilia B suffered from less accessibility to coagulation factor concentrates than patients with hemophilia A [11].

Previous studies on medical cost and hospital utilization for patients with hemophilia in mainland China mainly focused on medical expenditure or influencing factors for hospital utilization. For example, Song et al. analysed medical insurance costs and cost composition of different hemostatic agents by years, revealing that the majority of costs of hemostatic products was accounted for by pdFVIII, rFVIII and PCCs [12]. Gong et al. calculated the median medical expenditures on patients with hemophilia for the two urban basic medical insurance schemes [13]. There has been no research on the disparities in drug consumption and medical costs in terms of different types of hemophilia. This paper addresses these lacunae, assessing whether health costs and hospital utilization disparities existed between hemophilia A and hemophilia B inpatients, and, second, whether the prescription of coagulation factor concentrates for hemophilia A and hemophilia B inpatients was optimal, from the third payer perspective.

## Materials and methods

### Data sources

Between 2010 and 2016, a 5% random sample was extracted from the claims database of China Urban Employees’ Basic Medical Insurance (UEBMI) and Urban Residents’ Basic Medical Insurance (URBMI) by China Health Insurance Research Association (CHIRA). UEBMI and URBMI were the two main social health insurance schemes administered by Chinese government, covering more than 95% of the urban residents, for roughly 750 million, or 53%, of the total Chinese population in 2015 [14, 15]. Systematic random sampling strategy with a random start was adopted to collect the samples, where every Kth record from a population of size N was selected, with the first sample record picked from a random number table. In this way, a sample size of n was obtained, where *N/n> = *K [16]. Socio-demographic information, hospitalization costs and healthcare hospital utilization information on patients from all the 31 provinces in mainland China were also available in the database.

### Samples

Data for patients diagnosed with hemophilia defined by the ICD-10 code were extracted from the above sample database from January 2010 and December 2016. Hemophilia patients were identified if they had at least one inpatient claim with a primary diagnosis of hemophilia, with ICD-D66 referring to deficiency of clotting Factor VIII and ICD-D67 referring to deficiency of clotting Factor IX. Female patients were excluded to omit cases of von Willebrand disease [17]. Given the focus of previous research on inpatient costs as the main cost driver of medical expenditure in China, including hemophilia [13, 18, 19], and the inability to identify patients undergoing home treatment through our database, only information on hemophilia inpatients was extracted. Finally, 407 (83.57%) inpatients with hemophilia A and 80 (16.43%) inpatients with hemophilia B were included in our study. The prevalence of our random sample was consistent with nationwide one, which previously reported that hemophilia A accounted for 80-85% while hemophilia B accounted for 10-15% of the total hemophilia population in mainland China [20].

### Perspective of the study

Since the treatment and its cost related to hemophilia was covered by the UEBMI and the URBMI, the perspective of the third payer was used in this study.

### Method

Medical costs and hospital utilization rates allow disparities between hemophilia types to be tested. As recommended by guidelines from World Federation of Hemophilia (WFH), plasma-derived or recombinant FVIII concentrates (pdFVIII or rFVIII) should be the treatment of choice for hemophilia A, while FIX concentrates (including pure FIX concentrates or PCCs) should be the treatment of choice for hemophilia B [21, 22]. These different types of hemophilia patients allow us to easily distinguish by their drug type treatment whether drug prescription was optimal. It is not optimal when patients with hemophilia A and hemophilia B are prescribed exactly the same treatment. Medically, equivalent therapy approach to different types of hemophilia patients is not recommended. FVIII and FIX concentrates products with their brand names and unit cost (USD per international unit, calculated from the database) analyzed in our study are illustrated in Table 1.

We first compared the medical costs and hospital utilization between hemophilia A and hemophilia B using the Mann-Whitney test and multiple regression analysis to understand the economic burden of hemophilia by disease types in urban China. Then we reported and compared the usage and cost component of coagulation factor concentrates between hemophilia A and hemophilia B inpatients. Combined with guidelines from WFH and peer studies, it is possible to assess whether the delivery of coagulation factor products related to hemophilia was equitable and optimal in China’s real-world setting.

**Table 1:** Unit cost of coagulation factor concentrates with brand names

Coagulation factor concentrates		Brand name	Unit cost (USD/IU)
FVIII	Plasma-derived	AGCC^®^	0.3410
		CTBB^®^	0.2981
		HAMORAAS^®^	0.2981
		KANGSIPING^®^	0.2710
	Recombinant	ADVATE^®^	0.6305
		Kogenate^®^	0.6070
FIX	PCCs	KANGSHUNING^®^	0.1995

### Medical cost and hospital utilization estimation

As far as inpatient stays, costs were reimbursed according to a whole stay fee with a retrospective approach. Our data contained information on direct medical costs of hemophilia A and hemophilia B inpatients, categorized into medication costs and non-pharmacy costs. Medication costs referred to the costs of medicine intended to treat hemophilia or its complications, including hemostatic agents like clotting factor concentrates (pdFVIII, rFVIII, PCCs). Non-pharmacy costs referred to all other inpatient costs except medication costs, including diagnostic tests, non-medication therapy and medical consumables. We compared the healthcare expenditure between hemophilia A and hemophilia B in terms of total medical cost per patient, total medication cost per patient, total coagulation factor cost per patient and total non-pharmacy cost per patient. Hospital utilization was compared between hemophilia types in terms of number of hospitalizations per patient, length of hospital stay per patient. At last, a description of consumption and cost component of coagulation factor products by hemophilia types was presented with percentages.

Variables on inpatient characteristics (age and region), type of insurance (UEBMI, URBMI), city level (Class I, Class II, Class III), hospital level (primary, secondary, and tertiary) were also derived from the claims database for each year 2010-2016. Region comprised eastern, central and western provinces. The eastern region had the highest economic development level, followed by central and western region. According to administrative and economic status, city level was categorized as Class III provincial capital cities, Class II municipalities and Class I prefecture-level cities. Provincial capital cities and municipalities had more advanced medical resources compared with prefecture-level cities. Primary hospitals had less than 100 beds, providing basic health services to residents in a local community; secondary hospitals, with 100-500 beds, provided comprehensive health services to several communities as well as medical training and regional-based research; and tertiary hospitals, with over 500 beds, provided complex healthcare for several districts and undertook advanced medical education and research.

All costs were based on a constant 2016 US$1.0 = RMB 6.6423 annual average exchange rate. 

### Statistical analysis

Univariate analysis was conducted to compare medical cost and hospital utilization between hemophilia A and hemophilia B inpatients. Descriptive variables included age, region, city level, insurance type, hospital level, number of hospitalizations, length of stay, total inpatient cost per patient, total medication cost per patient, total coagulation factor cost per patient and total non-pharmacy cost per patient. Percentages of consumption and cost for different coagulation factor products were presented. Category variables were presented as absolute frequencies and percentages and tested by Pearson Chi-square test. The differences between medical cost and hospital utilization of hemophilia A and B inpatients were tested by the Mann-Whitney test based on median and interquartile range (IQR). Multiple regression analysis based on a generalized linear model was conducted, with logarithm of total inpatient costs as the dependent variable and hemophilia type as an independent variable, with age, number of hospitalizations, length of stay, region, city level, insurance type, hospital level, and years as control variables. A *P* value of less than 0.05 was considered statistically significant. Descriptive analysis and the Mann-Whitney test were performed using SPSS 24.0 for Window (IBM Corp., Armonk, NY, USA), and a multiple regression analysis was performed by STATA/SE 15.

## Results

### Patient characteristics

As shown in Table 2, a sample of 487 patients with hemophilia were identified, including 407 (83.57%) patients with hemophilia A and 80 (16.43%) patients with hemophilia B. All of the patients were male with mean age of 37.25 years old. Patients with hemophilia A (mean age of 36.42 years old) were significantly younger than patients with hemophilia B (mean age of 41.48 years old). Two hundred and five (42.1%) inpatients were from hospitals in eastern China, 157 (32.2%) were from central China hospitals and 125 (25.7%) from western region hospitals; 209 (42.9%) inpatients sought medical service in hospitals in Class III cities, while 86 (17.7%) inpatients were located in Class II cities and 192 (39.4%) inpatients in Class I cities. URBMI covered 263 (54.0%) inpatients, with 323 (66.3%) inpatients receiving medical treatment in tertiary hospitals, 133 (27.3%) in secondary hospitals and 31 (6.4%) patients in primary hospitals.

**Table 2:** Demographic characteristics and hemophilia patients in urban China 2010-2016

Characteristics		Overall (*n=*487)	Hemophilia A (*n=*407)	Hemophilia B *(n=*80)	*p*-value
Age, n(%)	<18	91(18.7)	76(18.7)	15(18.7)	0.015
	18-44	226(46.4)	194(47.7)	32(40.0)	
	45-64	111(22.8)	96(23.6)	15(18.8)	
	>64	59(12.1)	41(10.0)	18(22.5)	
Region, n(%)	Eastern area	205(42.1)	164(40.3)	41(51.2)	0.001
	Central area	157(32.2)	125(30.7)	32(40.0)	
	Western area	125(25.7)	118(29.0)	7(8.8)	
City level, n(%)	Class 3	209(42.9)	180(44.2)	29(36.3)	0.039
	Class 2	86(17.7)	64(15.7)	22(27.5)	
	Class 1	192(39.4)	163(40.1)	29(36.3)	
Insurance type, n(%)	UEBMI	224(46.0)	184(45.2)	40(50.0)	0.432
	URBMI	263(54.0)	223(54.8)	40(50.0)	
Hospital type, n(%)	Primary hospital	31(6.4)	26(6.4)	5(6.3)	0.839
	Secondary hospital	133(27.3)	109(26.8)	24(30.0)	
	Tertiary hospital	323(66.3)	272(66.8)	51(63.7)	
Year	2010	40(8.2)	22(5.4)	18(22.5)	0.001
	2011	34(7.0)	34(8.4)	0(0.0)	
	2012	44(9.0)	42(10.3)	2(2.5)	
	2013	72(14.8)	72(17.7)	0(0.0)	
	2014	56(11.5)	55(13.5)	1(1.3)	
	2015	106(21.8)	84(20.6)	22(27.5)	
	2016	135(27.7)	98(24.1)	37(46.3)	

*P* values are based on the chi-square test; *UEBMI*: Urban Employee Basic Medical Insurance scheme, *URBMI*: Urban Resident Basic Medical Insurance scheme

### Difference of direct medical cost and hospital utilization between hemophilia A and hemophilia B inpatients

Table 3 presents the direct medical cost and hospital utilization by hemophilia types. Patients with hemophilia B (USD 2912.81 / RMB 19347.76) spent significantly more on inpatient hospitalization expenses than hemophilia A (USD 1225.60 / RMB 8140.78, *P < *0.05) inpatients, and also had a significantly longer length of hospital stay (9.00 versus 7.00, *P < *0.05). Patients with hemophilia A and B had similar patterns of resource use, with both hemophilia types incurring roughly the same proportion of medical costs, with medication costs (85.85-92.24%) the largest percentage of total medical costs. As the largest share of medication cost, coagulation factor cost of patients with hemophilia B (USD 1073.12 / RMB 7128.00) was significantly higher than those with hemophilia A (USD 157.63 / RMB 1047.00, *P < *0.05). No statistical significance was observed in non-pharmacy cost and number of hospitalizations between two hemophilia types.

**Table 3:** Medical cost and hospital utilization for inpatients with hemophilia

Items		Hemophilia A	Hemophilia B	*p*-value
Total medical cost, RMB	Median	8140.78	19347.76	<0.001
	IQR	(2538.15-22635.83)	(7672.97-59978.5)	
Total medication cost, RMB	Median	4193.20	13700.44	0.001
	IQR	(583.7-17357.12)	(2738.10-59356.33)	
	% of total cost	85.85%	92.24%	
Total coagulation factor cost, RMB	Median	1047.00	7128.00	0.012
	IQR	(0-14220.8)	(0-53882)	
	% of total cost	76.86%	86.68%	
Non-pharmacy cost, RMB	Median	1301.06	1735.61	0.622
	IQR	(83-4090.6)	(0-5832.91)	
	% of total cost	14.42%	7.76%	
Number of hospitalizations, n	Median	1.00	1.00	0.259
	IQR	(1-2)	(1-5)	
Length of stay, days	Median	7.00	9.00	0.033
	IQR	(3-15)	(4-16)	

*P* values are based on the Mann-Whitney test; *IQR: *Interquartile range, *UEBM*I: Urban Employee Basic Medical Insurance scheme, *URBMI*: Urban Resident Basic Medical Insurance scheme

### Multivariate analysis of total inpatient costs between hemophilia types

To further model the difference in total inpatient costs by hemophilia types, Table 4 presents the results of the multiple regression generalized linear model. We found that hemophilia A inpatients had 41.7% (Coef.=-0.417, *P < *0.05) lower medical cost than hemophilia B inpatients, after adjusting for confounding factors, including age, number of hospitalizations, length of stay, region, city level, insurance type, hospital type and years of calendar.

**Table 4:** Multiple regression analysis of total inpatient costs

Parameters		Coef.	*P>*z	95% Wald confidence interval	
				Lower	Upper
Disease type (Reference: Hemophilia B)	Hemophilia A	-0.417	0.019	-0.764	-0.070
Age		-0.001	0.764	-0.008	0.006
Number of hospitalizations		0.092	0.000	0.067	0.117
Length of stay		0.004	0.005	0.001	0.007
Region (Reference: Western)	Eastern	0.768	0.000	0.416	1.120
	Central	0.560	0.003	0.191	0.929
City level (Reference: Class I)	Class III	0.354	0.018	0.061	0.646
	Class II	-0.065	0.749	-0.463	0.333
Insurance type (Reference: URBMI)	UEBMI	0.390	0.011	0.088	0.692
Hospital type (Reference: Primary)	Tertiary	0.651	0.009	0.166	1.136
	Secondary	0.280	0.295	-0.244	0.805
Year (Reference: 2010)	2011	0.716	0.010	0.171	1.262
	2012	0.603	0.026	0.073	1.133
	2013	0.755	0.014	0.155	1.355
	2014	-0.061	0.855	-0.722	0.599
	2015	0.249	0.425	-0.362	0.860
	2016	0.300	0.345	-0.322	0.923
Intercept		8.224	0.000	7.508	8.941

Parameter estimates from logged costs, *UEBMI*: Urban Employee Basic Medical Insurance scheme, *URBMI*: Urban Resident Basic Medical Insurance scheme

### Differences in coagulation factor concentrates for patients with hemophilia

**Fig. 1:**
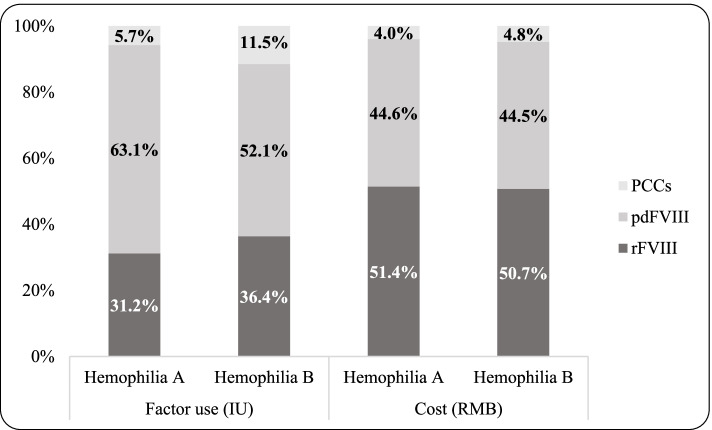
Usage and cost distribution of coagulation factor products by hemophilia types (percentages)

The results of Tables 3 and 4 reveal that there were significant differences in total inpatient costs as well as the length of stay between hemophilia A and hemophilia B. Table 3 shows that costs of coagulation factor products were the main cost drivers of total medical costs for both subtypes, with related costs accounting for dominant share of total costs (76.86-86.68%). Then what was the usage pattern of coagulation factor concentrates between hemophilia types? Figure 1 presents the consumption (IU, International Units) and cost (RMB) distribution of three types of coagulation factor products that were in use and covered by basic medical insurance schemes during our research period, by hemophilia types. For both hemophilia cohorts, the largest proportion of usage was plasma-derived Factor VIII (pdFVIII), accounting for 52.1-63.1% of total consumption of coagulation factor products, followed by recombinant Factor VIII (rFVIII), while patients with hemophilia B used larger share of prothrombin complex concentrates (PCCs) than those with hemophilia A (11.5% versus 5.7%). Regarding to the cost component, the percentages of rFVIII were larger than pdFVIII for both cohorts, mainly due to lower prices of pdFVIII products (see Table 1).

## Discussion

This is the first study to use Chinese health insurance claims data to compare the medical cost and hospital utilization, and to assess use of coagulation factor concentrates, between hemophilia A and B inpatients [23], from the third payer perspective. Consistent with previous studies [24–29], we found that the inpatient medical cost of hemophilia were mainly attributed to medication costs for both hemophilia A and hemophilia B inpatients. The cost of clotting factor concentrates accounted for the largest proportion of medical costs because hemophilia patients require lifetime treatment of expensive coagulation factor concentrates [30]. Hemophilia B inpatients bore significantly higher medical costs and medication expenses than hemophilia A inpatients, with medication costs for hemophilia B accounting for larger share of total medical expense than hemophilia A inpatients. This is the reverse of Yan et al. [31], who reported that hospitalized hemophilia A patients had significantly higher medical costs and medication costs than patients with hemophilia B in Taiwan. This discrepancy may be because the length of hospital stay of patients with hemophilia A in Taiwan was longer than patients with hemophilia B, while in our study patients with hemophilia B had significantly longer length of hospital stay. Previous studies indicated that longer length of stay and higher number of hospitalizations was associated with higher medical costs [15].

We estimated the distribution of consumption and cost of coagulation factor concentrates (CFCs) between hemophilia A and hemophilia B inpatients. With regard to the proportion of total inpatient cost, both subtypes consumed rFVIII the most, and PCCs the least, which was not only inconsistent with previous studies [24, 27, 32–35], but also contradictory to common clinical practice [36].

For patients with hemophilia B, pure FIX concentrates and PCCs should be optimal treatments of choice [37], resulting in high consumption of pure FIX concentrates and PCCs. But we found that recombinant and plasma-derived FVIII concentrates, which should not be prescribed to hemophilia B patients, were broadly used among patients with hemophilia B. While hemophilia B inpatients received lower levels of PCCs use. We inferred that wastage of FVIII concentrates and suboptimal prescription of coagulation factor concentrates were incurred by hemophilia B inpatients, which might account for the additional coagulation factor cost bore by hemophilia B inpatients to some extent. It should be noted that within our study period of 2010-2016, pure FIX concentrates (plasma-derived and recombinant FIX concentrates), which have been proved to reduce risk of thrombosis and disseminate intravascular coagulation compared to PCCs [22], were not covered by the basic medical insurance schemes, so their costs were not able to be estimated. In addition, we found that besides FVIII concentrates, PCCs were also prescribed to patients with hemophilia A. This could be because inpatients with hemophilia A with inhibitors, the most severe complication of hemophilia [38], have been treated with PCCs as bypassing agents to stop acute bleeding [39]. Such utilization of PCCs for hemophilia A is consistent with a real-world study in China, which reported substantial usage rate of PCCs by patients with hemophilia A with inhibitors (76.2%) and limited options for hemostatic agents among them [39]. But, in terms of best-practice hemophilia A with inhibitors treatment, PCCs were much less effective treatment choice than rFVIIa. rFVIIa was also not covered by the basic medical insurance schemes until 2017, which meant rFVIIa relevant data were not analyzed in our study.

According to a report by WHO, the lack of prompt, appropriate treatment in hemophilia may lead to prolonged hospitalization and the misuse or wastage of expensive blood products [40]. We can infer from our results that there was a suboptimal use of coagulation factor concentrates, with the subsequent higher medical cost and hospital utilization for hemophilia patients, which was especially critical for those with hemophilia B, imposing a cost on the health system as well as individual hemophilia patients.

There are some factors influencing the decision-making when choosing hemostatic products in clinical practice, such as physician bias and insurance coverage [41]. Although the Hemophilia Treatment Center Collaborative Network of China (HTCCNC) now has expanded to 120 clinics throughout the country since its establishment in 2004, they are mostly in tertiary urban hospitals. Patients in remote areas usually seek medical treatment in non-HTCCNC hospitals, especially during times of acute bleeding. As a result, inpatients without access to HTCCNC hospitals were likely to obtain less timely and expert hemophilia-related healthcare services. Also, medical insurance coverage in China is complex and varies across different geographical regions, with reimbursement rates for hemophilia ranging from 40–94% [42]. Lower reimbursement rates may lead to lower willingness for physicians and patients to utilize expensive products and insufficient use of drugs [42].

Healthcare system reforms in China might complicate matters further. Before 2009, influenced by the drug mark-up policy and bonus system, physicians were rewarded based on the monetary values of drugs they prescribed, say, 15% profit margin for drug sales [43], resulting in over-prescribing and high drug prices for patients [44]. As the biggest reform of China’s health system implemented in 2009, the zero-markup drug policy removed the profit margin from drug sales and increased the prices for medical services that need labour input [44]. Under the circumstances, many hospitals are reluctant to store and provide adequate clotting factor concentrates to control the share of drug expenditure to total cost [11], because they are often associated with high costs and few patients. This situation may be even worse for patients with hemophilia B with fewer populations. The administrative sector should secure the provision of coagulation factor concentrates and eliminate all barriers to drug accessibility for hemophilia patients. Therefore, patients with hemophilia in mainland China may experience a high economic burden and disease risk with limited insurance coverage and accessibility for drugs.

The paper has several limitations. First, patients’ medical data, such as body weight, treatment patterns (prophylaxis vs. on-demand), complications, severity of disease, number of vital bleeds and quality of life were not available in the claim dataset, which meant that we can not identify patients with inhibitors through laboratory testing. These missing variables might impact the results. Second, data of rural residents with hemophilia and outpatient visits are not included in our analysis. Future studies need to collect outpatient and rural data. Third, our study only covers the 2010-2016 period in mainland China, with post**-**2016 health care reform likely to impact our results. Despite these limitations, our study clearly identified disparities in medical cost and hospital utilization between hemophilia A and hemophilia B in urban China and found use of coagulation factor concentrates was suboptimal.

## Conclusions

For the first time, this study explored the disparities of hospital cost and medical utilization between hemophilia A and hemophilia B using the basic medical insurance claims database for urban China. Patients with hemophilia B experienced significantly higher inpatient cost and coagulation factor cost than patients with hemophilia A. Our findings revealed the suboptimal use of coagulation factor concentrate drugs and the higher economic burden incurred by hemophilia B inpatients. Our results suggest that additional progress in the management of hemophilia in China is required and physicians managing hemophilia patients should adhere to the current World Federation of Hemophilia (WFH) guidelines and best hemophilia practice. Our results also have implications for hemophilia disease management, especially the use of coagulation factor concentrates, for the developing world.

## Data Availability

The data that support the findings of this study are available from China Health Insurance Research Association but restrictions apply to the availability of these data, which were used under license for the current study, and so are not publicly available. Data are however available from the authors upon reasonable request and with permission of China Health Insurance Research Association.

## Abbreviations

UEBMI: Urban Employees’ Basic Medical Insurance;
URBMI: Urban Residents’ Basic Medical Insurance;
IQR: Interquartile Range;
IU: International Units;
PCCs: Prothrombin ComplexConcentrates
